# Presentation of Preauricular Sinus and Preauricular Sinus Abscess in Southwest Nigeria

**Published:** 2013-12

**Authors:** W. A. Adegbiji, B. S. Alabi, O. A. Olajuyin, C. C. Nwawolo

**Affiliations:** 1 Department of ENT, University of Ado-Ekiti teaching hospital, Nigeria;; 2 Department of ENT, University of Ilorin teaching hospital, Nigeria;; 3 Department of ENT, Lagos University teaching hospital, Lagos, Nigeria

**Keywords:** Preauricular sinus, preauricular sinus abscess, congenital malformation

## Abstract

**BACKGROUND AND AIM:**

Preauricular sinus abscess is a common congenital external ear disease. This abscess is usually misdiagnosed because it is commonly overlooked during physical examination. In Nigeria, the prevalence was 9.3% in Ilorin, north central Nigeria This study is to determine the distribution and clinical presentation of the preauricular sinus abscess in Ekiti, south west Nigeria.

**MATERIALS AND METHODS:**

This is a prospective hospital based study of all patients with diagnosis of preauricular sinus abscess seen in our clinic carried out between April 2008 to March 2010. Detailed clinical history, administered interviewer’s assisted questionnaires full examination and. Data obtained were collated and analysed.

**RESULTS:**

Preauricular sinus were noticed in 184 (4.4%) out of 4170 patients seen during the study period. Preauricular sinus abscess were noticed in 21 (11.4%) of the preauricular sinuses especially in children. Unilateral preauricular sinus abscess accounted for 90.5%.

Common presenting complaints were preauricular swelling (81.0%), 90.5% with recurrent earaches, 76.2% with ear discharges. All patients had antibiotic / analgesic while 17 out of 21 (81.0%) had surgical excisions.

**CONCLUSION:**

Preauricular sinus abscess were noticed among 11.4% of the preauricular sinuses especially in children, unilateral preauricular sinus abscess accounted for 90.5%. Common complaints were otorrhoea, earaches, and swelling and they were mostly managed surgically.

## INTRODUCTION

Preauricular sinuses are congenital malformations that usually occur at anterior margin of the ascending limbs of the helix of the external ear (1-3). They are not rare anomalies of the ear though they are not frequently diagnosed.

Preauricular sinus occurs with different prevalent rates among blacks and Caucasians. In different parts of the world, the prevalence varies, in the USA it is 0.1-0.9%, England is 0.9%, Taiwan is 1.6-2.5%, among Asian, it occurs in 4-6% of the population and some parts of Africa it is 4-10% (4-9). In Nigeria, the prevalence was 9.3% in Ilorin, northern Nigeria.

Hereditary play part in its distribution but it may be sporadic (10-12). Developmentally, the external ear develops from six eminences on the mandibular and hyoid margin of the first external groove. Failure of the tubercles to fuse with each other or failure of some of these tubercles (hillock) to grow normally may produce a variety of external ear malformation such as congenital preauricular sinus (2, 3, 13). Preauricular sinus is usually asymptomatic unless it is infected.

Preauricular sinuses are prone to infection leading to preauricular sinus abscess, when it infected, it is mainly by Staphylococcus aureus and less commonly by Streptococcus and Proteus (10). These results in irritation, fluid drainage, oedema, pain and when the sinus ostium is blocked pus accumulate leading to abscess formation. It may also be complicated by spreading to contiguous structures such as the pinna, temporomandibular joint and external auditory canal.

Clinical presentations of preauricular sinus abscess are usually recurrent ear discharge, pain, swelling, itching, headache and fever. Other congenital anomalies such as hearing loss or renal problem of 1.7% and 2.6% respectfully are usually associated with preauricular sinus (1).

Limitation to the diagnosis and effective treatment of preauricular sinus abscess are failure to diagnose. If not infected it is frequently asymptomatic and may be omitted on routine ear examination (1). Most symptomatic patients with preauricular sinus abscess usually seek no medical attention probably because it is less troublesome and lack of awareness of its presence, also infrequently mentioned in medical literature. For these reasons most physicians may be unaware of its existence.

Preauricular sinus abscess is commonly mistaken for pimples (blackheads), furunculosis, chronic infection such as tuberculosis and fungal also congenital condition such as dermoids and sebaceous cysts (14).

Like in other parts of the world, there is paucity of literature on preauricular sinus abscess in this environment. This study aimed at determines the distribution and clinical presentation of preauricular sinus abscess among Nigerians at Ekiti, south west Nigeria.

## MATERIALS AND METHODS

This is a prospective hospital based study of all patients seen in Ear, Nose and Throat Clinic of the University Teaching Hospital, Ado Ekiti, capital of Ekiti State, Nigeria. It is the main referral hospital for the Ekiti community of south western Nigeria.

The study was carried out between April 2008 to March 2010 after ethical approval from the hospital ethical committee and informed consent was taken from patients or their guardian before their enrollment into the study.

All patients with clinical diagnosis of preauricular sinus abscess were enrolled into the study, interviewer’s assisted questionnaires were administered to all the subjects and detail histories of presenting complaints were taken.

Detailed otologic, nasal and throat examinations were performed on all the enrollees. Full general examinations were done to rule out associated congenital anomalies.

Hearing assessment including pure tone audiometry and tympanometry were performed to assess the quality and quantity of hearing aquity. In addition, abdominopelvic ultrasound scan was performed to rule out congenital renal anomalies.

Limitation to this study was only patient seen in Ear, Nose and Throat clinic of our clinic excluding other clinics.

Data obtained were collated and statistically analysed descriptively using SPSS version 11 computer software. Data were expressed by using tables, bar and pie charts.

## RESULTS

A total of 4170 patients were seen over the study period and 184 (4.4%) subjects had preauricular sinus. Preauricular sinus abscess were noticed in 21 (11.4%) subjects. Prevalence of preauricular sinus was 4.4% while prevalence of preauricular sinus abscess was 0.5%. Female accounted for 57.1% and male 42.9.

Majority of preauricular sinus abscess were symptomatic during childhood with 42.9% of those found to be equal to or less than 10 years at age (Table 1).

**Table 1:** Age Distribution of Patients with Preauricular Sinus Abscess

Age Range	Number	Percentage (%)

1-10	9	42.9
11-20	5	23.8
21-30	7	33.3
Total	21	100

Unilateral preauricular sinus abscess was found in 90.5% and was bilateral in 9.5%. 13 (61.9%) occurred on the right ear while 7 (28.6%) occurred on left ear. Familial history and finding of preauricular sinus was noticed in 23.8%. Greater than 50% of the subjects have an average of three recurrent episodes per year.

Common presenting complaints were 81% preauricular swelling, 90.5% recurrent ear pain, 76.2% recurrent ear discharge and 52.4% itching (Table 2). No associated renal anomalies or hearing impairment were recorded.

**Table 2:** Clinical complaints of preauricular sinus abscess

Clinical features	Percentage occurrence (%)

Recurrent ear pain	90
Swelling front of the ear	80
Recurrent ear discharge	75
Ear itching	50
Headaches	30
Fever	20

**Figure 1:**
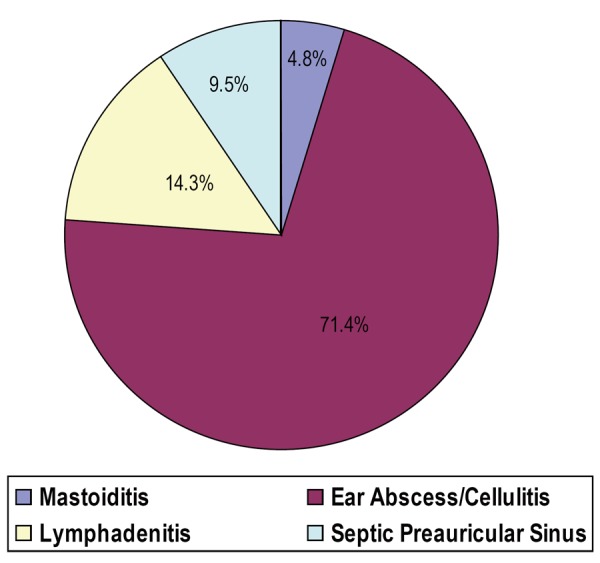
Diagnosis made by referring Physicians. All our studied patient with preauricular sinus abscess were treated with antibiotic and analgesic to treat acute infection and pain. Seventeen (81.0%) had surgery, which are excisional biopsy in 3 (14.3%) and incision with drainage in 16 (76.2%) to relief abscess. Recurrent cases were noticed to be high and it depend on treatment modalities. This was 95.2% with antibiotic, 68.8% with incision and drainage and 0% excisional biopsy.

**Figure 2:**
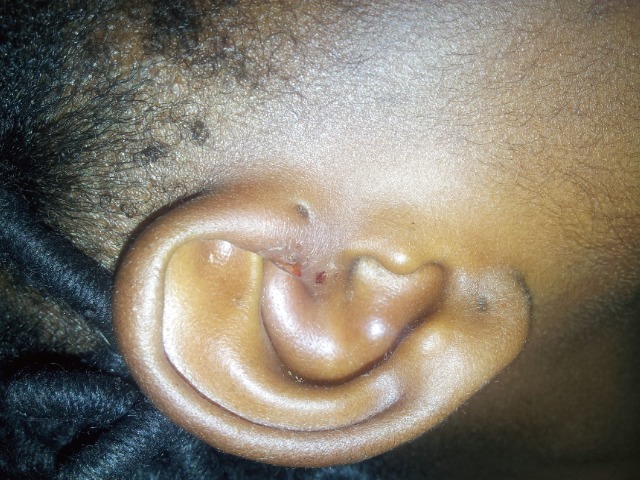
Preauricular sinus abscess of the right ear.

From Figures 1, diagnosis by referring physician were ear abscess/cellulitis, lymphadenitis, mastoiditis and preauricular sinus abscess in 71.4%, 14.3%, 4.8% and 9.5% respectively. Prior to presentation patient had received various forms of treatment on the preauricular sinus abscess. These were 100% antibiotic/analgesic; 71.4% herbal medication such shrubs and concoctions; 23.8% incision and drainage while 17 out of 21 (81.0%) had surgical excisions. Associated complications of preauricular sinus abscess were 85.7% discomfort, 42.9% cellulitis, 14.3% perichondritis, 3.3% hypertrophic scar and 9.5% keloid formation. Figure 2 shows preauricular sinus abscess of the right ear.

## DISCUSSION

Preauricular sinus is a congenital malformation, which usually manifest during childhood or early in life as in majority of our studied patients. Majority of congenital anomalies were known to be symptomatic during childhood. As in other studies, preauricular sinus abscess was predominantly found in children in our study (1). It may be as a result of more attention parents give to their children’s health condition.

This congenital malformed ear tubercle leaves a blind tube which once infected, will habour infected agent. This is responsible for high rate of infection of preauricular sinus and abscess formation, 11.4% in this study similar to Jimoh et al study in Ilorin, Nigeria.

The preauricular sinus abscess affects both sexes in this study like in other studies. The proportion varies from different studies and race. Some studies show that men and women were equally affected (15-17). Some works support women to be predominantly affected (18-21). Jimoh *et al* study revealed a male preponderance. This study revealed high proportion of preauricular sinus abscess in female. This may be due to facial make up and cosmetic usage in women.

Preauricular sinuses, once infected are prone to frequent and recurrent infection hence prior to presentation more than 50% of the subject had three episodes per year. This is due to residual bacteria in the sinus and susceptibility of the preauricular sinus to infection. Also recurrent preauricular sinus abscess in our study was related to the prior treatment modalities. Complete surgical sinus excision eradicate residual sinus bacterial while medications do not result in complete bacteriological cure.

Affectation of the right ear is commoner in our study than the left. This may be because many people are of right handedness than left handedness. So right ear are more probed than left ear. Other studies also establish commoner unilateral and higher right ear cases (3, 20).

Common clinical presentations of this condition are discharge, erythema, and preauricular swelling (1). This finding is similar to our finding of swelling in front of ear, recurrent ear discharge and earache. Single or recurrent infection leads to the complication findings in this study such as ear discomfort, cellulitis, perichondritis, hypertrophic scar as well as keloid.

In our study unlike other studies, no case of hearing impairment or renal disorder was detected. This may be due to the number of cases studied. Some other studies also revealed syndromes association with preauricular sinus (21, 22). Hearing impairment was found to be 8/1000 among infants with preauricular skin tags or ear pits in a study done by Daphne *et al* (23).

Common errors are made in the diagnosis of preauricular sinus abscess. This could be due to scanty report and low information in the literature on preauricular sinus abscess. This could also be due to higher percentage of asymptomatic cases (14). As in this study, very low percentage of the subject had accurate diagnosis made and surgical option remains the treatment for preauricular sinus abscess once infections are controlled after recurrent episodes.

## CONCLUSION

Preauricular sinus abscess is a congenital condition of the external ear, is common but not usually noticed until symptomatic or complicated preauricular sinus abscess were noticed among 11.4% of the preauricular sinuses especially in children, unilateral preauricular sinus abscess accounted for 90.5% while right preauricular sinus abscess accounted for 61.9%. Common complaints were ear discharge, ear pain and swelling and they were mostly managed surgically. There are few reported cases of preauricular sinus abscess in the literatures hence the need to increase level of awareness.

## References

[1] Tan T, Constantidines H, Mitchell TE (2005). The preauricular sinus: A review of its aetiology, Clinical presentation and management. Int. J. Pediatr. Otorhinoillaryngol.

[2] Lawrence MD, Tom WC, Daniel MD (2002). Surgical treatment of preauricular sinus/cysts. Paediatr. Otolaryngol.

[3] Scheinfeld NS, Silverberg NB, Weinberg JM (2004). The preauricular sinus: A review of its clinical presentation treatment and associations. Pediatr. Dermatol.

[4] Jimoh OR, Alabi BS, Adebayo SS (2008). Prevalence of preauricular sinus among Nigerians. Surgery Journal.

[5] Adam M, Hudgins L (2003). The importance of minor anomalies in the evaluation of the newborn. Neo Reviews.

[6] Huang XY, Tay GS, Wansaicheong GK, Low WK (2007). Preauricular sinus. Clinical course and associations. Arch. Otolaryngol. Head Neck Surg.

[7] Lizama M, Cavagnaro F, Arau R, Navarrete O (2007). Association of isolated preauricular tags and nephrourological anomalies: Case-control study. Pediatr. Nephrol.

[8] Deshpande SA, Watson H (2006). Renal ultrasconography not required in babies with isolated minor ear anomalies. Arch. Dis. Child Fetal Neonatal. Ed.

[9] Firat Y, Sireci S, Yakinci C, Akarcay M (2008). Isolated preauricular pit and tags: Is it necessary to investigate renal abnormalities and hearing impairment. Eur. Arch. Otorhinolaryngol.

[10] Amarilis Sanchez-Valle, Xueqing Wang, Lorraine Potocki, Zhilian Xia (2002). HERV-Mediated Genomic Rearrangement of EYA1in an Individual with Branchio-oto-renal Syndrome. Eur. J. Hum Genet.

[11] Bellini C, Piagglo G, Massocco D, Perfumo FB (2001). Brachio-oto-renal syndrome. A report on nine family groups. Am. J. Kidney Dis.

[12] Zou F, Peng Y, Wang X, Sun A (2003). A locus for congenital preauricular fistula; maps to chromosome. 8q 11.1 – q 13.3. J. Hum. Genet.

[13] Alfred DK (1965). Preauricular sinuses: A congenital heredity anomaly. Am. J. Surg.

[14] Yeo SW, Jun BC, Park SN, Lee JH (2006). The preauricular sinus: factors contributing to recurrence after surgery. Am. J. Otolaryngol.

[15] Chang PH, Wu CM (2005). An insidious preauricular sinus presenting as an infected postauricular cyst. Int. J. Clin Pract.

[16] Gur E, Yeung A, Al-Azzawi M, Thomson H (1998). The excised preauricular sinus in 14 years experience: Is there a problem?. Plast. Reconstr. Surg.

[17] Baatenburg de Jong RJ (2005). A new surgical technique for treatment of preauricular sinus. Surgery.

[18] Currie AR, King WW, Vlantis AC, Li AK (1996). Pitfalls in the management of preauricular sinus. Br. J. Surg.

[19] Lam HC, Soo G, Wormald PJ (2001). Excision of the preauricular sinus: A comparison of two surgical techniques. Laryngoscopes.

[20] Paulozzi LJ, Lary JM (1999). Laterality patterns in infants with external birth defects. Teratology.

[21] Sardana K, Sharma RC, Jain A (2002). Facial steotocytoma multiplex associated with pillar cyst and bilateral preauricular sinus. J. Dermatol.

[22] Petersen MB, Hansen M, Djernes BW (1987). Full trisomy 22 in a newborn infant. Annal. Genet.

[23] Roth DA, Hildesheimer M, Bardenstein S, Goidel D (2008). Preauricular skin tags and ear pits are associated with permanent hearing impairment in newborns. Pediatr.

